# Setting health research priorities using the CHNRI method: V. Quantitative properties of human collective knowledge

**DOI:** 10.7189/jogh.06.010502

**Published:** 2016-06

**Authors:** Igor Rudan, Sachiyo Yoshida, Kerri Wazny, Kit Yee Chan, Simon Cousens

**Affiliations:** 1 Centre for Global Health Research, The Usher Institute for Population Health Sciences and Informatics, the University of Edinburgh, Edinburgh, Scotland, UK; 2 Department for Maternal, Newborn, Child and Adolescent Health, World Health Organization, Geneva, Switzerland; 3 Nossal Institute for Global Health, University of Melbourne, Melbourne, Victoria, Australia; 4 Department of Infectious Disease Epidemiology, London School of Hygiene and Tropical Medicine, London, UK

## Abstract

**Introduction:**

The CHNRI method for setting health research priorities has crowdsourcing as the major component. It uses the collective opinion of a group of experts to generate, assess and prioritize between many competing health research ideas. It is difficult to compare the accuracy of human individual and collective opinions in predicting uncertain future outcomes before the outcomes are known. However, this limitation does not apply to existing knowledge, which is an important component underlying opinion. In this paper, we report several experiments to explore the quantitative properties of human collective knowledge and discuss their relevance to the CHNRI method.

**Methods:**

We conducted a series of experiments in groups of about 160 (range: 122–175) undergraduate Year 2 medical students to compare their collective knowledge to their individual knowledge. We asked them to answer 10 questions on each of the following: (i) an area in which they have a degree of expertise (undergraduate Year 1 medical curriculum); (ii) an area in which they likely have some knowledge (general knowledge); and (iii) an area in which they are not expected to have any knowledge (astronomy). We also presented them with 20 pairs of well–known celebrities and asked them to identify the older person of the pair. In all these experiments our goal was to examine how the collective answer compares to the distribution of students’ individual answers.

**Results:**

When answering the questions in their own area of expertise, the collective answer (the median) was in the top 20.83% of the most accurate individual responses; in general knowledge, it was in the top 11.93%; and in an area with no expertise, the group answer was in the top 7.02%. However, the collective answer based on mean values fared much worse, ranging from top 75.60% to top 95.91%. Also, when confronted with guessing the older of the two celebrities, the collective response was correct in 18/20 cases (90%), while the 8 most successful individuals among the students had 19/20 correct answers (95%). However, when the system in which the students who were not sure of the correct answer were allowed to either choose an award of half of the point in all such instances, or withdraw from responding, in order to improve the score of the collective, the collective was correct in 19/20 cases (95%), while the 3 most successful individuals were correct in 17/20 cases (85%).

**Conclusions:**

Our experiments showed that the collective knowledge of a group with expertise in the subject should always be very close to the true value. In most cases and under most assumption, the collective knowledge will be more accurate than the knowledge of an “average” individual, but there always seems to be a small group of individuals who manage to out–perform the collective. The accuracy of collective prediction may be enhanced by allowing the individuals with low confidence in their answer to withdraw from answering.

In 1906, Galton suggested that a group of individuals make better predictions as a collective than any individual expert [1]. Since then, our understanding of the “Wisdom of Crowds” has grown: in recent years, a widely appreciated example of this phenomenon has been evident to the audience of the quiz show “Who Wants To Be A Millionaire?” In this quiz show, a contestant needs to answer a series of increasingly difficult questions by picking from one of four possible responses, only one of which is correct – so that the probability that a random response is correct is 25%. In this show, an “*Ask the audience*” joker is available, whereby 100 persons in studio audience get to submit electronically their opinion on what the correct answer is, and the distribution of their individual opinions is then shown to the contestant. As an alternative, a “*Phone a friend*” joker allows contestants to phone one friend whom they consider the most knowledgeable, and then ask for his/her individual answer. Comparative analyses of the performance of the two jokers showed that the relative majority of the audience chose the correct answer about 91% of the time, while the most knowledgeable friend was right about 65% of the time. There are methodological concerns over the direct comparison between these two percentages, because these success rates were based on different questions, but the difference is still quite striking [1].

Crowdsourcing has become an increasingly popular human tool to address many problems–from government elections in democracies [2], formation of stock market prices [3], to modern online platforms such as TripAdvisor (to advise on the best hotels and restaurants) [4] or Internet Movie Database (IMDb) (to advise on the best movies, TV shows, etc.), all of which are based on the personal opinions of many hundreds or thousands of participants [5]. When crowdsourcing is used for gathering information, or in decision–making processes, there is probably a need to distinguish between at least three different scenarios in which collective knowledge might be used. The first is getting the right answer to a factual question, which we may consider “objective knowledge” and it represents the simplest case. The second is predicting the outcome of some future event, which can subsequently be verified with certainty and within a reasonable time frame. An example is betting on an outcome, eg, of football games or horse races. This is different from stock market predictions, where those who participate in predictions (investors) can also influence the outcomes through their actions. Finally, crowdsourcing could be used to gather information on subjective opinion on something that cannot be easily verified. This last scenario is the closest to how crowdsourcing is used in the CHNRI method (the acronym for: Child Health and Nutrition Research Initiative) [6,7], which seeks to gauge collective optimism with respect to different health research ideas and the benefits they might lead to at some point in the future.

The CHNRI method for setting health research priorities uses “crowds” of experts in global health – researchers, policy makers and programme implementers – to generate, assess and prioritize between many competing ideas in global health research. A CHNRI exercise produces a ranking of many research ideas according to the collective opinion of the expert group, but it is not possible to verify objectively how “valid” that ranking may be, not least because low ranked ideas are unlikely to be funded and therefore no outcomes are available for them. It is yet to be demonstrated that the collective opinion of an expert group should be regarded as more useful than the opinion of individual experts in the group [1,8]. However, the difficulties related to validating personal opinions do not apply to the validation of personal knowledge, and the accuracy of personal knowledge is an important component underlying the individual’s opinion. Because of this, we should expect some parallels between the quantitative properties of human collective knowledge and human collective opinion. In this paper, we report several experiments to explore the quantitative properties of human collective knowledge and discuss their possible relevance to the validity of the CHNRI method. The aim of this paper is to examine the accuracy of collective compared to individual knowledge, using different approaches of assessment.

## METHODS

We conducted a series of experiments among a group of undergraduate medical students. The number of participating students ranged from 122 to 175 in each exercise. Students who completed the second year lectures in Epidemiology and Statistics, as part of a practical application of epidemiological and statistical concepts were asked to answer 10 questions on each of the following: (i) an area in which they have a degree of expertise (subjects related to the medical curriculum for the first year undergraduate); (ii) an area in which they have some knowledge but do not have expertise (general knowledge); and (iii) an area in which they are not expected to have any knowledge (astronomy). The content of the lecture was entirely unrelated to the questions that were asked from the students. The ethics approval was obtained from a relevant research centre (Centre for Population Health Sciences at the University of Edinburgh).

The questions were chosen so that the answer to each question was numerical (an integer), and so that the answers ranged from a 1–digit number to a 10–digit number over the course of 10 questions in random order, with students unaware of this element of the design. This element was included to allow us to assess whether the students’ answers were more accurate when the correct answer was a smaller or larger number (see **Online Supplementary Document(Online Supplementary Document)**).

**Table 1** shows the questions that were asked in each of the three areas, and the correct answers. The questions were asked at the end of 3 consecutive lectures spanning 10 days. Students were given 30 seconds to answer each question. The students were asked to record an answer for every question. For questions for which they were unsure of the answer they were asked to write down their best guess.

**Table 1:** Questions posed to a group of undergraduate Year 2 medical students*

**Questions in an area of students’ high expertise (undergraduate Year 1 medical curriculum)**	
1. How many valence electrons does carbon have?	(4)
2. How many pairs of cranial nerves are there?	(12)
3. How many bones in the adult human body?	(206)
4. In which year did Freud publish “The interpretation of dreams”?	(1900)
5. How many genes does a human have?	(23 000)
6. What is an average salary of a GP in the UK?	(104 000)
7. How many erythrocytes in 1 mL of blood?	(5 000 000)
8. How many refugees are there in the world?	(15 400 000)
9. How many people in the world have diabetes?	(347 000 000)
10. How many bases (A, T, C or G letters) are in the haploid human genome?	(3 000 000 000)
**Questions in an area of students’ moderate expertise (general knowledge)**	
1. How many marriages did Elizabeth Taylor have?	(8)
2. How old was Mozart when he died?	(35)
3. How many minutes does the movie “Casablanca” last?	(102)
4. In which year was Hamlet first published?	(1603)
5. How many diseases in ICD–10?	(14 400)
6. What is the average house price in the UK (in GBP)?	(238 976)
7. How many people live in Cape Town?	(3 740 000)
8. How much was Van Gogh’s “sunflowers” painting sold for (in US$)?	(39 700 000)
9. What is the population size of Indonesia?	(246 900 000)
10. How many views did Psy’s “Gangham Style” video have to date?	(1 764 039 000)
**Questions in an area of student’s low expertise (astronomy)**	
1. How many light years from our Sun is Sirius?	(9)
2. How many moons does Saturn have?	(62)
3. How many times is Jupiter heavier than Earth?	(318)
4. In which year was Uranus first discovered?	(1781)
5. Distance between our Sun and the centre of Milky Way galaxy (in light–years)?	(27 000)
6. How many times is the Sun heavier than Earth?	(332 900)
7. What is the speed of the solar wind (in Km/h)?	(1 440 000)
8. How many years ago did the comet impact killed off dinosaurs?	(65 000 000)
9. Distance between the Sun and the Jupiter (in kilometres)?	(780 000 000)
10. How many years ago was our Solar System formed?	(4 568 000 000)

*The group was about 170 (range: 167–175) undergraduate Year 2 medical students from: (i) an area of their high expertise (ie, undergraduate Year 1 medical curriculum); (ii) an area where they have some expertise (general knowledge); and (iii) an area where they should have no expertise (astronomy). Correct answers are shown in brackets.

In addition, students were shown 20 pairs of well–known celebrities and asked them identify which was the older of the two. **Table 2** shows the pairs of celebrities in the order that the questions were asked. The questions were phrased as: “*Would you say that Celebrity X is older than Celebrity Y?*”, and the possible answers were either “*Yes*” or “*No*”, where they had to choose one of those two options. However, they were also given an option next to each answer to choose their “secondary” answer as either “*Not sure*” (when they were familiar of both celebrities, but it was too difficult to judge), or leaving the answer “*Blank*” deliberately, when not knowing one or both celebrities. Those two options would indicate their low confidence in their “Yes”/”No” answer. By adding “*Not sure*” (which would be coded with half a point) or “*Blank*” (which would remove them from the sample, leaving the others with more confidence in their answers), they could prevent a wrong answer and increase the chance of the collective answer to be close to the correct answer. This latter type of “scoring” is also used by the CHNRI method. In this way, the same group of students provided two different data sets with scores: one, where they all needed to provide a binary (“Yes”/”No”) answer to each question, regardless of their confidence in answering the question correctly; and the other one, where they were able to use the answer “*Not sure*”, or leave the answer blank, when they were not confident in their answer. Their input was then turned into a data sheet that was analogous to those produced in the CHNRI exercise, where “*Yes*” was coded as “1”, “*No*” as “0”, “*Not sure*” as “0.5” and “*Blank*” responses were simply left as blank cells in the data sheet.

**Table 2:** Questions posed to a group of 122 undergraduate medical students to guess which well–known celebrity is older than the other*

Pair 1: Justin Bieber vs Miley Cyrus (19 vs 20)
Pair 2: George Clooney vs Brad Pitt (52 vs 49)
Pair 3: Madonna vs Susan Boyle (55 vs 52)
Pair 4: Beyonce vs Shakira (32 vs 36)
Pair 5: Dustin Hoffman vs Robert de Niro (76 vs 70)
Pair 6: Katy Perry vs Rihanna (28 vs 25)
Pair 7: Mick Jagger vs Paul McCartney (70 vs 71)
Pair 8: Lewis Hamilton vs Tiger Woods (28 vs 37)
Pair 9: Angela Merkel vs J. K. Rowling (59 vs 48)
Pair 10: Tony Blair vs George W. Bush (60 vs 67)
Pair 11: David Cameron vs Barack Obama (47 vs 52)
Pair 12: Ashton Kutcher vs Ben Affleck (35 vs 41)
Pair 13: Tom Cruise vs Nicole Kidman (51 vs 46)
Pair 14: Paris Hilton vs Jennifer Anniston (32 vs 44)
Pair 15: Jennifer Lopez vs Britney Spears (44 vs 31)
Pair 16: Eminem vs Jay–Z (40 vs 43)
Pair 17: Kim Kardashian vs Adele (33 vs 25)
Pair 18: Roger Federer vs Andy Murray (32 vs 26)
Pair 19: David Beckham vs Prince Harry (38 vs 29)
Pair 20: Elvis Presley vs Michael Jackson (42 vs 50)

*Correct answers (expressed in years of their age at the time of this exercise) are shown in brackets. The indicated age of individuals is relevant to October 17, 2013. For the last pair, the age at the time of death was being compared. The question was posed as: “Would you say that celebrity X is older than celebrity Y?” and possible answers were “Yes”, “No”, “Not sure” or “Blank” (see details in the text).

This design was carefully developed to allow us to study two questions: (i) how the students’ collective opinion performs in comparison to that of individuals when the answers are no longer in a quantitative, but rather in a categorical format; and (ii) whether the *format* of categorical answer (with or without allowing for “*Not sure*” when students’ confidence in their answer is low, or “*Blank*” when they simply don’t have any knowledge on the question) altered the performance of the students’ collective answer. Our hypothesis was that allowing students to answer “*Not sure*” or “*Blank*” would give better results, because it allows the participants within a team who are not sure of the correct answer to “withdraw” from providing their (possibly inaccurate) input, which would give more weight to the responses from students who were more confident in their individual knowledge.

Thus, four different experiments were conducted over the course of four consecutive lectures, which we label “Medical knowledge–quantitative” (MKQ), “General knowledge–quantitative” (GKQ), “Astronomy knowledge–quantitative” (AKQ) and “Celebrity knowledge–categorical” (CKC). In the MKQ, GKQ and AKQ exercises, we conducted the analyses in the following way: (i) we determined the median and the mean response for each of the 10 questions, based on all answers collected from the students (sample sizes were N = 167, N = 175 and N = 170, respectively); (ii) we also developed a parameter that we called “error size”, to quantify the extent to which each student deviated from the correct answers over a series of 10 questions, and then we also applied it to the collective median and mean. Given that the responses could both over– or under–estimate the true value, we were interested in the ratio between the larger and the smaller of the two (ie, the correct answer and the answer provided by the student). As an example, this means that, if the correct answer was “10”, and one student provided the answer “2” and the other “50”, they would be making errors of the “same size”: in our evaluation, it was equally wrong to over– or underestimate some value 5–fold. This also means that if the correct answer was provided for each question, then all the ratios contributing to “error size” parameter would be “1”. Any deviation from the correct answer in either direction would increase the parameter from this theoretical minimum. (Note that this differs from other possible approaches, such a proportionally expressed increase or decrease, because the latter system would favour under–estimation as a smaller error than over–estimation, and under–estimation would be limited to 100% while overestimation would not be limited in any way). Once the individual errors, expressed as the ratio of the greater vs the smaller of the two values, was determined for each answer to each question, they were summarized for each individual student across all 10 questions and their sum was called “error size”. In this way, each student was assigned his/her own “error size” in each of the three exercises (GKQ, MKQ and AKQ), and the students were then ranked by the error size parameter, from the smallest to the largest error made. This was then repeated for the entry of a collective (both using medians and means), and median and mean value rank within the entire student sample was then determined.

In the fourth exercise (CKC), which we designed as a series of 20 “*Yes* or *No*” questions, the task for the students was changed. In the first instance, the collective answer was taken to be the answer given by the majority of students–either “*Yes*” or “*No*”. Then, there was an additional methodological caveat. First, those who were not confident about their answer could change some of their answers into the “*Not sure*” option, the effect of which contributed a certain 0.5 points to a total score, and minimised the risk of dropping a whole point for the collective for an incorrect answer. Second, those who had no knowledge of the question (eg, not recognising the names of celebrities) were allowed to change some of their responses to “*Blank*”. This would have the effect of reducing the sample size of the collective, leaving all those with no knowledge out, and reducing the overall threshold of correct answers required from other students that the collective would need to answer correctly. Clearly, for those who are confident of their knowledge, this system would mean that they should answer “*Yes*” or “*No*” to all questions and not use either “*Not sure*” or “*Blank*” options at all.

The *correct* answer was then coded as “1”, “*not sure*” as “0.5”, the *incorrect* answer as “0”, and “*blanks*” were excluded from the analysis, thus reducing sample size. The points assigned as described above were added (“1” for correct, “0.5” for “*not sure*”, and “0” for incorrect) and then divided by the total number of “*non–blank*” responses received. The result was expressed as “*the percentage of correctness*” of the collective answer, and any value greater than 50% was considered a correct collective answer. This produced two data sheets–CKC1 (where everyone was required to submit either a *Yes* or a *No* answer) and CKC2 (with a *Yes–No–Not sure– Don’t know scoring system*). The comparison between the two exercises was expected to reveal if “self–removal” through the use of “*Not sure*” or “*Blank*” improves the score of the collective considerably.

## RESULTS

Students’ collective answers (median and mean) to the 10 questions in three areas: (i) an area of their expertise, ie, Year 1 medical curriculum; (ii) the area of general knowledge; and (iii) the area outside of their expertise, ie, astronomy are shown in **Tables 3 to 5** (a total of 167, 175 and 171 responses received, respectively). **Table 6** shows the summary result of the three exercises, presenting both the rank and the percentile of the collective answer (based on either median or mode) among all individual answers provided by the students in three consecutive exercises where students had a decreasing level of expert knowledge. When answering the questions in their own area of expertise, the collective numerical median answer was 35/168 (21^st^ centile) of the most accurate answers; in general knowledge, it was 21/176 (12^th^ centile) most accurate answers; and in an area with no expertise, the group answer was the 12/171 (7^th^ centile). However, the mean value of the collective didn’t rank highly in any of the three exercises–in fact, it ranked near the bottom: 127/168 (76^th^ centile) in Year 1 medical knowledge, 164/176 (93^rd^ centile) in general knowledge and 164/171 (96^th^ centile) in astronomy.

**Table 3:** Year 2 undergraduate medical students’ collective answers to the 10 questions in the area of their knowledge*

Question	Correct answer	Students’ collective answer–median	Students’ collective answer–mean
1. Valence electrons in carbon?	4	4	6
2. Number of cranial nerve pairs?	12	12	13
3. Number of bones in human body?	206	206	210
4. Freud’s “Interpretation of dreams” published?	1900	1901	1890
5. Number of human genes?	23 000	38 000†	1 124 128 437
6. Average GP’s salary in the UK?	104 100	76 001	85 568
7. Erythrocytes in 1 mL of blood?	5 000 000	8 679	12 124 582
8. Number of refugees in the world?	15 400 000	80 000 000	394 267 469
9. Number of people with diabetes?	347 000 000	100 000 000	444 785 232
10. Number of ATCGs in human genome?	3 000 000 000	23 500 327	178 090 845 668

*Number of responses N = 167.

†Question 5 was problematic because the number of human genes was revised down from about 40 000 to 23 000 only recently, ie, after the students learned of the former number; therefore, the median response from students was, in fact, very close to what they were likely to have learnt earlier in the course of their education).

**Table 4:** Year 2 undergraduate medical students’ collective answers to the 10 questions in the area of general knowledge*

Question	Correct answer	Students’collective answer (median)	Students’ collective answer–mean
1. Number of marriages of Elizabeth Taylor?	8	4	4
2. How old was Mozart when he died?	35	38	40
3. Minutes duration of “Casablanca”?	102	120	122
4. Year when “Hamlet” was published?	1603	1642	1637
5. Number of diseases in ICD–10?	14 400	48 132	76 480 054
6. Average house price in the UK?	238 976	193 271	369 819
7. Population size of Cape Town?	3 740 196	3 000 000	19 384 089
8. Price of van Gogh’s “Sunflowers”?	39 700 000	15 000 000	3 875 825 789
9. Population size of Indonesia?	246 900 000	20 000 000	682 312 629
10. Number of views of “Gangnam Style”?	1 764 039 000	278 000 000	1 610 122 583

*Number of responses N = 175.

**Table 5:** Year 2 undergraduate medical students’ collective answers to the 10 questions in the area outside of their expertise (astronomy)*

Question	Correct answer	Students’ collective answer (median)	Students’ collective answer (mean)
1. Distance Earth–Sirius (in light–years)?	9	6900	5 800 659 084
2. Number of Saturn’s moons?	62	12	20
3. How many times Jupiter heavier than Earth?	318	811	5 681 716 865
4. When was Uranus first discovered?	1781	1807	1720
5. Distance Sun–Milky Way Centre (in ly)?	27 000	5 000 000	22 584 267 640
6. How much Sun heavier than Earth?	332 900	8 000	8 561 716 703
7. Speed of Solar Wind (in km/h)?	1 440 000	43 027	7 948 573 823
8. Years since comet killed off dinosaurs?	65 000 000	24 564 456	1 396 252 256
9. Kilometres from Sun to Jupiter?	780 000 000	8 728 001	1 239 338 648 469
10. Years since solar system created?	4 568 000 000	7 119 851 052	721 049 090 361

* Number of responses N = 170.

**Table 6:** The rank and the percentile of the collective answer (based on either median or mean) among all individual answers provided by the students in three consecutive exercises where students had a decreasing level of expert knowledge*

	Collective answer–median			Collective answer–mean		
**Exercises on collective knowledge**	**Rank**	**Percentile (% top answers)**	**“Error size” parameter**	**Rank**	**Percentile (% top answers)**	**“Error size” parameter**
Medical (Year 1) knowledge	35/168	20.83%	725	127/168	75.60%	48 975
General knowledge	21/176	11.93%	38	164/176	93.18%	5430
Astronomy knowledge	12/171	7.02%	1132	164/171	95.91%	663 265 715

*Addition of the collective answer increased the total number of received answers by one, resulting in 168, 176 and 171 responses being ranked in each exercise, respectively; percentile of eg, 20.83% means that the collective response ranked among the 20.83% most accurate individual responses).

**Table 7** shows the results of the exercise in recognizing the older of the two celebrities, based on the sample of 122 participating students. The age indicated in the table was relevant to October 17, 2013. All 20 questions were phrased as: “*Would you say that Celebrity X is older than Celebrity Y*?” The possible answers in the first round were “Yes” or “No” (2–category system); and in the second round the students were also allowed “Not sure” (when they were familiar of both celebrities, but it was too difficult to judge) and leaving the answer “Blank” deliberately (when not knowing one or both celebrities), in order to increase the chance of the entire collective of students to answer correctly. The latter type of “scoring” is used in the CHNRI method.

**Table 7:** Results of the exercise in recognizing the older of the two celebrities (N = 122)*

Older celebrity	Younger celebrity	Difference (years)	% correct (2–category system: yes/no)	% correct (4–category system: yes/no/ns/b)
Roger Federer (32)	Andy Murray (26)	6	97%	97%
George Clooney (52)	Brad Pitt (49)	3	95%	96%
David Beckham (38)	Prince Harry (29)	11	96%	96%
Tiger Woods (37)	Lewis Hamilton (28)	11	93%	95%
Jennifer Aniston (44)	Paris Hilton (32)	12	97%	94%
Miley Cyrus (20)	Justin Bieber (19)	1	93%	92%
Ben Affleck (41)	Ashton Kutcher (35)	6	85%	85%
George W. Bush (67)	Tony Blair (60)	7	85%	80%
Kim Kardashian (33)	Adele (25)	8	82%	79%
Jennifer Lopez (44)	Britney Spears (31)	13	83%	78%
Angela Merkel (59)	JK Rowling (48)	11	71%	73%
Michael Jackson (50)	Elvis Presley (42)	8	75%	67%
Barack Obama (52)	David Cameron (47)	5	66%	62%
Tom Cruise (51)	Nicole Kidman (46)	5	64%	60%
Katy Perry (28)	Rihanna (25)	3	63%	59%
Jay–Z (43)	Eminem (40)	3	56%	57%
Dustin Hoffman (76)	Robert de Niro (70)	6	44%	52%
Paul McCartney (71)	Mick Jagger (70)	1	59%	52%
Madonna (55)	Susan Boyle (52)	3	55%	51%
Shakira (36)	Beyonce (32)	4	43%	43%

*The questions were phrased as: “Would you say that Celebrity X is older than Celebrity Y?”. The possible answers in the first round were “Yes” or “No” (2–category system); and in the second round the students were also allowed “Not sure” (when they were familiar of both celebrities, but it was too difficult to judge) and leaving the answer “Blank” deliberately (when not knowing one or both celebrities), in order to increase the chance of the entire collective of students to answer correctly. The latter type of “scoring” is used in the CHNRI method.

The results show that, when everyone needed to provide a “Yes” or “No” answer, regardless of their confidence in their own answer, the collective was correct in 18/20 cases (90%), with 8 students outperforming the results of the collective–all of them with 19/20 correct answers (95%). This means that the collective answer based on this type of response ranked in the top 7.3% of individual answers. However, when the students were allowed to use the system of responses in which those who were not confident of their answer were allowed to ask for half a point, or withdraw from responding entirely, in order to improve the scores of the collective, the results changed somewhat. Looking at all specific celebrity pairs, they were not clearly better than when everyone gave an answer regardless of their confidence in being correct. However, with this type of scoring the collective was correct in 19/20 cases (95%), while the 3 most successful individuals among the 122 students now had 17/20 correct guesses (85%). This clearly shows that many students opted to only receive half a point, or withdrew, because the small group among them who gave best individual answers did not repeat the level of success from the first round of scoring in this second round–although they did manage to further improve the collective answer. A subsequent analysis showed that the median frequency of choosing the “*Not sure*” answer when this was possible was 44 (range: 3–59), or about one third of students, with very wide range–depending on the level of difficulty of the question. The option “blank” was used much less frequently, with a median of 7 (range: 0–35).

The **Online Supplementary Document(Online Supplementary Document)** presents several additional analyses. Figures S1–S3 show that the number of digits of the correct answer does not seem to be related to the likelihood that the group will identify the correct answer–this only seemed to possibly be the case in the exercise where students had expertise (ie, Year 1 medical curriculum questions), but was not replicated in the other two exercises. Figure S4, related to the fourth exercise, shows that the proportion of those guessing correctly in the group was associated with the age difference between the two celebrities, as might be expected.

## DISCUSSION

The analyses conducted in this study tried to provide insights into quantitative properties of human collective knowledge, many of which are relevant to better understanding of the properties of the CHNRI method as originally proposed. First, the CHNRI method relies on the opinion of experts that is based on their knowledge of a specific subject, and asks them to express their optimism about research ideas through scores. Through this series of exercises we wanted to explore if this approach is likely to result in better predictions than if persons with limited knowledge of the subject are also invited to prioritize health research, or if persons with no knowledge at all are invited. In the student exercise in their own area of expertise (Year 1 medical curriculum, **Table 3**), the first 5 answers given by the students as a collective median value were all exactly right or extremely close (taking into account that the number of genes in the human genome was indeed close to 40 000 in their earlier textbooks, and it was only revised down to about 23 000 more recently). This level of precision was not observed in their responses to general knowledge questions (**Table 4**), or questions on astronomy (**Table 5**).

However, there are worrying signs that, when the majority of students don’t know the correct answer to a question that should be covered by their expert knowledge, the collective median can be very wrong. The examples are the case of the number of erythrocytes in 1 mL of blood (where the collective median was 3 orders of magnitude smaller than the correct value) or the number of nucleotides in the human genome (where the underestimate was by 2 orders of magnitude) (**Table 3**). Because of those two questions, where most of the students didn’t even know the right order of magnitude, the parameter “error size” of the collective median was even greater for the exercise on Year 1 medical knowledge, than it was for the exercise in general knowledge (**Table 6**). Although this may seem surprising at first, it can be easily explained. The parameter “error size” is very sensitive to the size of the departure from each of the 10 correct answers. In general knowledge questions, collective median answers were always reasonably close to the correct answers in terms of students’ being able to guess the correct order of magnitude for the answer, as all the questions were related to topics in which they had at least some knowledge. However, a specific question in their own area of expertise in which they had no knowledge could quickly lead to very large departures from the correct answer. It would be difficult, given a small sample size, to reach a definite conclusion that there are some experts who do better than the crowd–”*the superforecasters*” [8], although this remains a possibility.

The exercise in the knowledge of astronomy (**Table 5**) was interesting because it clearly showed that humans do not possess a “cryptic” ability to collectively predict values on which they do not have any knowledge as individuals with any precision. This suggests that “wisdom of crowds” only works when the majority of participants in the group have at least some private knowledge of the quantity that is being predicted. As an example, the students had some intuition on the possible year when Uranus could have been discovered, the number of Saturn’s moons, or even the number of years since the Solar system was created–they got the order of magnitude correct in those three questions. However, when asked about quantities of which they knew nothing, nor had any intuition, they were typically wrong by several orders of magnitude when their collective medians were compared to the correct answers.

Collective medians typically performed well across all three exercises: the collective median was among the 20.83% of the most accurate responses in the medical knowledge, 11.93% in the general knowledge, and 7.02% in the astronomy knowledge. We propose that the collective median is actually not among the top 10% scores in the area of expertise, because there is a smaller group of students among the entire cohort with excellent knowledge, and who would be seen as the top of their class. These students know the correct answers and the rest of the class simply dilutes their accuracy and moves the collective median away from the perfectly accurate response. We believe that this explains why the collective median in the area of expertise was only at the 21^st^ percentile of the most accurate answers. However, as the collective moves towards answering the questions outside of the area of their expertise, the collective median begins to move up the ranks. Once there are no longer individuals who could easily answer all 10 questions with high accuracy, the collective median progresses to the 12^th^ percentile (in the general knowledge exercise) and 8^th^ percentile (in astronomy exercise).

We propose a mathematical explanation for this, which is relevant to the relationship between the correct answer and the distribution of all responses in a series of questions. After each question, the collective median will be exactly at the 50^th^ percentile of answers. When the distribution of answers is compared to the correct answer, the error size of the median will either be at the 50^th^ percentile of the group or smaller. For individual students who don’t have any knowledge on the subject and are simply guessing, they can expect to alternate between a position above and below the 50^th^ percentile randomly, and occasionally making gross mistakes. After enough time and many iterations, the collective median of a group who are guessing entirely unknown quantities will always be either at the 50^th^ percentile, or above, while the rest of individual answers will be above or below the 50^th^ percentile half of the time. After a sufficient number of questions, this should ensure that the collective median acquires Rank 1, because median can sometimes be very close to a correct answer, but never worse than 50^th^ percentile of all group’s guesses. This protects it from gross errors that all other students will eventually experience over a large number of guesses. This may be a general mechanism that explains why collective median eventually outperforms individuals in a long time series of predictions of entirely unknown quantities.

All of the above is relevant to collective medians. Turning our attention to collective means, they did not fare well at all. They were at the 76^th^ percentile of ranks in the area of medical knowledge, 94^th^ in the area of general knowledge, and 96^th^ in the area of astronomy. We found the explanation to this poor performance in a number of extremely wrong predictions made by several individuals, who made mistakes of such magnitude that they completely dominated the collective mean. Because of this, we suggest that – when the answers are being predicted in a quantitative form – medians will be more reliable than the means. One question that could be raised here is whether the entire cohort of medical students can be trusted to take this sort of exercise seriously, because if a small group deliberately put down extreme responses, this would certainly have an effect of skewing the mean.

The exercise in “guessing the older of the two celebrities” allowed us to establish that, in an area of “relative” expertise (because it has become difficult to avoid information on the celebrities that were chosen). There is considerable accuracy in collective prediction when “Yes”/”No” answers are allowed and the answer given by the majority is chosen as the correct one. The collective was correct in 90% of cases, and this translated to the rank 9/123 (8^th^ percentile in the ranks), with 8 individuals who recorded 95% of correct answers and out–performed the collective. This exercise was analogous to a large extent to the “Ask the audience” joker that is used in the quiz show “Who wants to be a millionaire?”, as mentioned earlier, and the accuracy of 90% is very similar to the one of about 91% observed in the quiz show.

The key question in this exercise was whether the collective response could be further improved by allowing some individuals, who were not confident in their answers, to minimise the “damage” to the collective by choosing “not sure” (which still gives them a guaranteed 50% of available points) or to drop out from the sample. When this option was given, the accuracy of the collective answer increased to 95%, while the three best individual answers only achieved 85%. A question–by–question comparison of 20 individual answers between the two types of scoring doesn’t indicate that the collective answer with the 2^nd^ type of scoring (4 options) is consistently better than the binary “Yes”/”No” type of scoring, so we cannot be sure that this finding is generalizable, rather than a chance effect, and we should continue to explore this with more questions and using larger sample sizes to confirm it.

We will now consider how the findings of this study are relevant to “validation” of the CHNRI method. This study shows that the collective knowledge in an area of expertise is likely to lead to more accurate responses than the collective knowledge in an area outside of the expertise. Moreover, the exercise shows that it may be better to only invite a reasonably small, highly selected group of experts and rely on their collective prediction, rather than trying to seek expertise from a large group, which may lead to deviations from the optimal collective prediction. This justifies the strategy that has been used in many early CHNRI exercises, where as few as 10–15 leading experts in a narrow research field were invited to conduct the exercise on setting research priorities in their field. Moreover, the type of response used in CHNRI exercises (“Yes” – ”No” – ”Not sure” – ”Blank”) seems to slightly improve the collective prediction in comparison to the alternative, where all scorers are forced to choose between only two binary options. However, the difference between the two types of scoring resulted in predictions that could be considered surprisingly similar, so further experiments will need to resolve whether there is a real difference between the two approaches or not. If there is no difference, then perhaps the “Yes”/”No” answer could be preferred as simpler and more discriminative in the process of prioritisation, because too many “not sure” answers lead to scores that show regression to the mean and the discriminatory power of the scoring process is gradually lost. This, therefore, remains an unresolved question that warrants further investigation.

Applications of “crowdsourcing” are finding ways into many areas of human activity. In parallel, many interesting scientific experiments are being performed to improve our understanding of the principles underlying and governing crowdsourcing. Recent studies showed that sharing the information on confidence in their answers between the individuals in the group can substantially improve the prediction of the group, as we could see in our study (**Table 7**), but if those most confident are wrong, then it can also lead the collective opinion to dramatically wrong decisions [8,9]. Independence of the provided opinion, such as in the CHNRI exercise, is very important because studies have convincingly shown that interactions between participants in the group and social influence may both improve and undermine the “wisdom of crowds” effect [10,11]. We should also mention that this research was conducted in “artificial”, well–controlled conditions, but in the real world every group will have its own unique dynamics. In many contexts, collective knowledge, opinion or intelligence may not be the main factor influencing the decisions, which is a limitation of this type of research and of its applications in complex real–world scenarios.

There seems to be agreement between researchers that select groups of “best–performing” experts can reach an optimal collective result with sample sizes as small as five, which cannot be easily improved by increasing sample size [12,13]. This observation has a potential practical application in the field of medical diagnostics [13]. However, it has also been shown that a well–designed mathematical or statistical model would still outperform any collective human opinion [13]. Two further interesting applications of crowdsourcing in the fields of medicine and health research have been proposed recently. One study proposed that, in the absence of clear guidelines on indications, stabilization of the prevalence of use of certain drugs–such as antidepressants–at the level of the whole population might indicate the optimal usage. This is because the stabilized frequency at the population level is likely to reflect hundreds of thousands of decisions on continued usage, made by treated individuals based on their personal experiences [14]. Finally, it has been proposed that complex, expensive and bureaucratic processes of research evaluations, such as the Research Excellence Framework (REF) that takes place every 6 years in the UK, could be replaced by crowd–sourced “prediction markets” [15]. Prediction markets enable individuals to trade “bets” on whether a specific outcome would occur or not, and they have been shown to be successful at predicting outcomes in different areas of human activity, such as sport, entertainment and politics. Given that they are based on expert judgements, which also form the basis of REF in the UK, there is no reason why prediction market could not theoretically offer an alternative to the REF that could be updated annually, or even track the performance in real time [15].

## CONCLUSION

Our experiments showed that the collective knowledge of a group with expertise in the subject should always be very close to the true value. In most cases and under most assumptions, the collective knowledge will be more accurate than the knowledge of an “average” individual, but there always seems to be a small group of individuals who manage to out–perform the collective. The accuracy of collective prediction may be enhanced by allowing the individuals with low confidence in their answer to withdraw from answering. This study showed that the CHNRI method is based on the premises and designs that are likely to maximise the predictive value of the group: experts are being invited to score proposed research ideas (instead of persons with limited knowledge, or lay persons); experts are providing their answers independently (to protect the end result from social influences); and they are using the scoring system that is expected to maximise the accuracy of the collective answer over the individual ones.

## References

[1] Surowiecki J. The wisdom of crowds. London: Abacus, 2013.

[2] Surowiecki J. Democracy: dreams of the common good. In: Surowiecki J. The wisdom of crowds. London: Abacus, 2013.

[3] Graham B. The intelligent investor. New York: Harper Collins, 2003.

[4] Tripadvisor. Available at: https://www.tripadvisor.co.uk/. Accessed: 15 April 2016.</eref>

[5] Internet Movie Database. Available at: http://www.imdb.com/. Accessed: 15 April 2016.

[6] Rudan I, Chopra M, Kapiriri L, Gibson J, Lansang MA, Carneiro I (2008). Setting priorities in global child health research investments: universal challenges and conceptual framework.. Croat Med J.

[7] Rudan I, Gibson JL, Ameratunga S, El Arifeen S, Bhutta ZA, Black M (2008). Setting priorities in Global child Health Research Investments: guidelines for implementation of the CHNRI Method.. Croat Med J.

[8] Tetlock PE, Gardner D. Superforecasting: the art and science of prediction. London: Random House Books 2015.

[9] Koriat A (2012). When are two heads better than one and why?. Science.

[10] King AJ, Cheng L, Starke SD, Myatt JP (2012). Is the true “wisdom of the crowd” to copy successful individuals?. Biol Lett.

[11] Lorenz J, Rauhut H, Schweitzer F, Helbing D (2011). How social influence can undermine the wisdom of crowd effect.. Proc Natl Acad Sci U S A.

[12] Mannes AE, Soll JB, Larrick RP (2014). The wisdom of select crowds.. J Pers Soc Psychol.

[13] Kattan MW, O’Rourke C, Yu C, Chagin K (2016). The wisdom of crowds of doctors: their average predictions outperform their individual ones.. Med Decis Making.

[14] Patten SB (2015). The wisdom of crowds (vox populi) and antidepressant use.. Clin Pract Epidemiol Ment Health.

[15] Munafo MR, Pfeiffer T, Altmejd A, Heikensten E, Almenberg J, Bird A (2015). Using prediction markets to forecast research evaluations.. R Soc Open Sci..

