# Supplementary material for: Setting health research priorities using the CHNRI method: V. Quantitative properties of human collective knowledge
Source: J Glob Health. 2016 Jun 20;6(1):010502. doi: 10.7189/jogh.06.010502 (PMC4920010; doi:10.7189/jogh.06.010502)

Online Supplementary Document

Rudan et al. Setting health research priorities using the CHNRI method: V. Quantitative properties of human collective knowledge

JoGH 2016;6:010502

SUPPLEMENTARY ONLINE MATERIAL

Figure S1. The association between the number of digits in the correct answer (x-axis) and the "error size" parameter (ratio, Y-axis) of a collective median answer in the exercise on medical Year 1 knowledge.

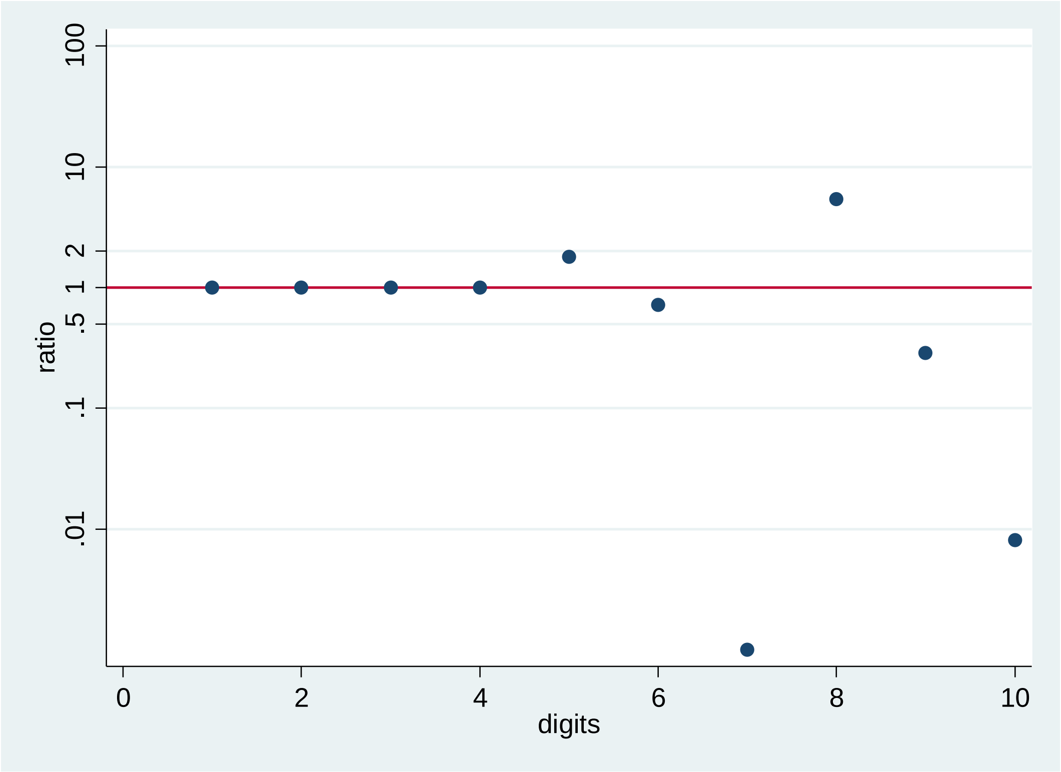

Figure S2. The association between the number of digits in the correct answer (x-axis) and the "error size" parameter (ratio, Y-axis) of a collective median answer in the exercise on general knowledge.

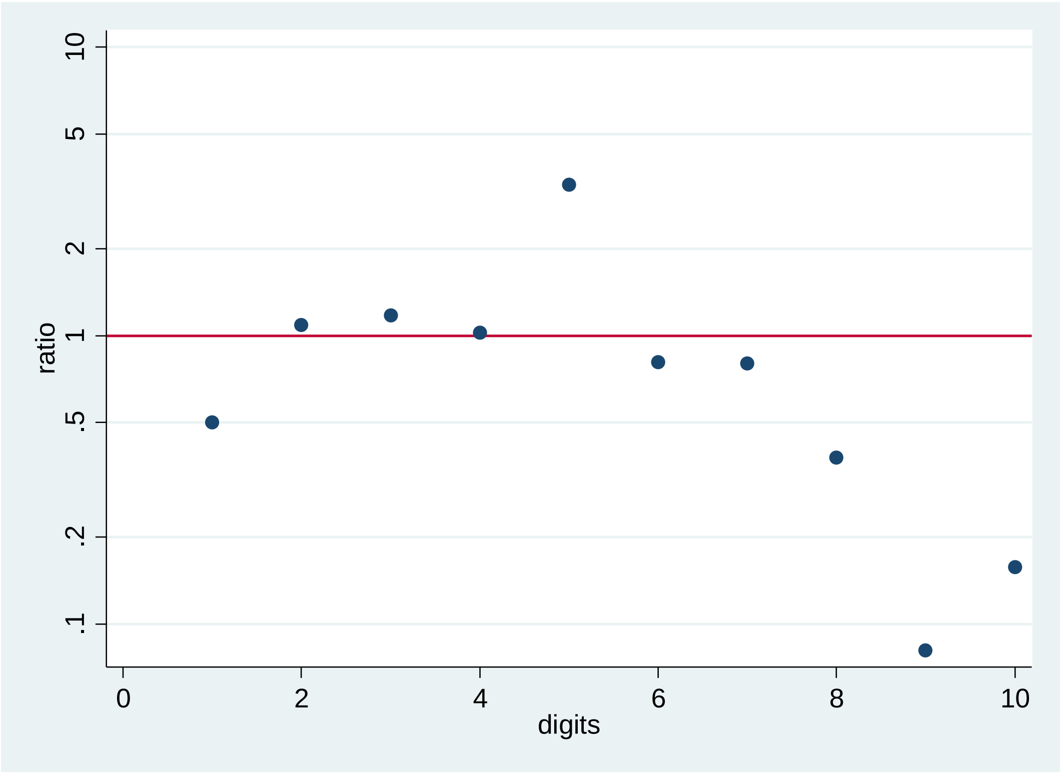

Figure S3. The association between the number of digits in the correct answer (x-axis) and the "error size" parameter (ratio, Y-axis) of a collective median answer in the exercise on astronomy knowledge.

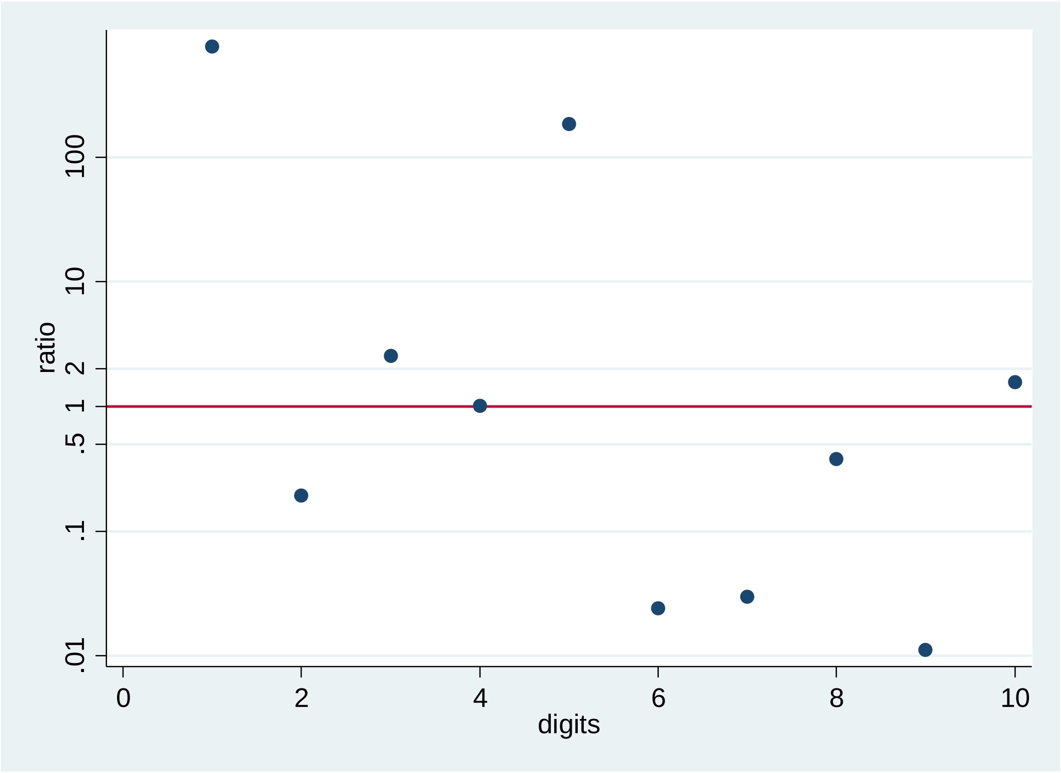

Figure S4: The association between the actual age difference (in years, x-axis) and percentage of positive answers in the exercise of guessing the older celebrity in a pair of celebrities.

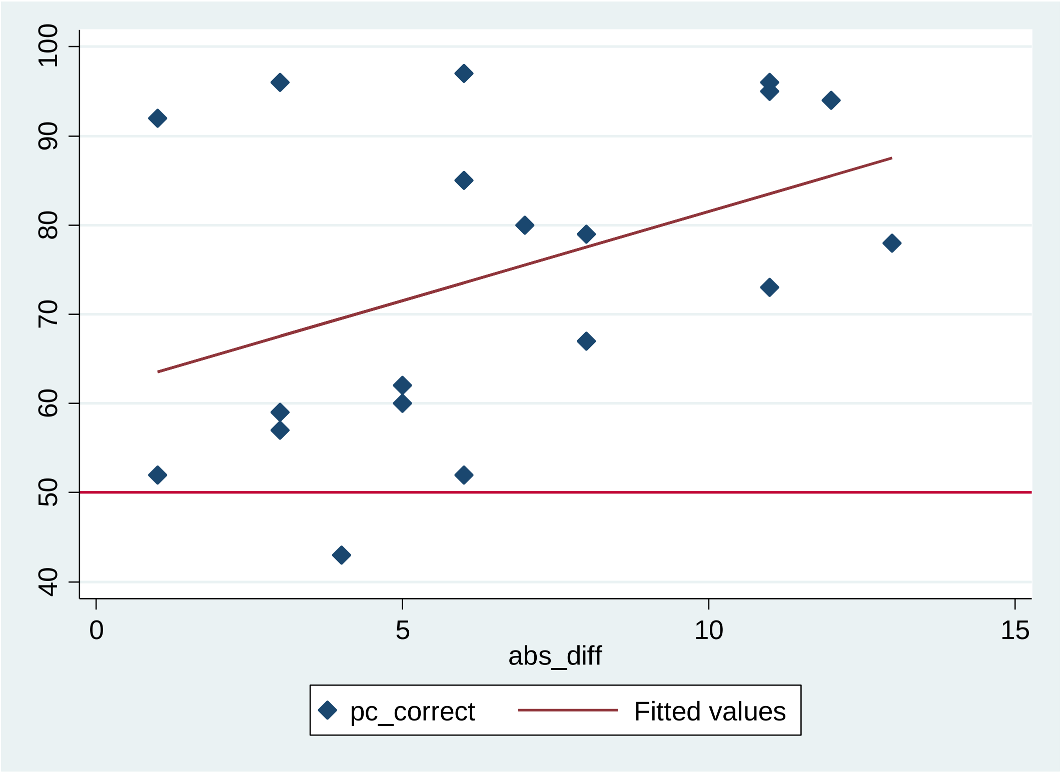

Supplement: Online Supplementary Document [file jogh-06-010502-s001.pdf]
